# Bibliometric analysis of research progress in pediatric intussusception from the Web of Science Core Collection over the past 15 years

**DOI:** 10.1097/MD.0000000000048054

**Published:** 2026-03-13

**Authors:** Jialin Chen, Xiaoting Wang, Dong Xu, Yong Du, Ping Yang

**Affiliations:** a Department of Clinical Medicine, North Sichuan Medical College, Nanchong, Sichuan Province, People’s Republic of China; b Department of Pediatric Surgery, Suining Central Hospital, Suining, Sichuan Province, People’s Republic of China; c Southwest Medical University, Luzhou, Sichuan Province, People’s Republic of China.

**Keywords:** bibliometrics, development trends, intussusception, pediatric surgery, research hotspots, rotavirus vaccine

## Abstract

**Background:**

The aim of this study was to systematically investigate the knowledge structure, historical trends, and emerging research frontiers in the domain of pediatric intussusception through bibliometric and visualization methods.

**Methods:**

A comprehensive search was performed in the Web of Science Core Collection to retrieve articles published from 2010 to 2024. VOSviewer and CiteSpace were utilized for bibliometric analysis, including co-occurrence networks, keyword clustering, and burst term detection. Additionally, the PubMed database was utilized to supplement and analyze clinical research trends in the field of intussusception.

**Results:**

After rigorous screening, a total of 993 relevant articles were included, contributed by 1580 institutions and 3702 authors (comprising both first and corresponding authors). The institution with the highest publication output was the US Centers for Disease Control and Prevention (CDC), while the most prolific author was Professor Umesh D. Parashar, also from the Centers for Disease Control and Prevention. The United States was the leading country in terms of publication volume, followed by China. These studies were published across 288 journals, with the Journal of Pediatric Surgery being the most prolific. Keyword clustering revealed 8 primary research themes: rotavirus vaccine, appendicitis, hydrostatic reduction, double-blind, Henoch–Schönlein purpura, epidemiology, small bowel intussusception, and Peutz–Jeghers syndrome. Clinical research on intussusception is predominantly composed of retrospective cohort studies (33.1%), followed by randomized controlled trials (26.3%). Key research focuses include intussusception treatment, management, diagnosis, and safety evaluation of rotavirus vaccines.

**Conclusion:**

This study provides a comprehensive overview of pediatric intussusception research over the past 15 years, highlighting a shift in focus from basic clinical observations to evidence-based practices and precision medicine. Future research should prioritize interdisciplinary collaboration, the development of personalized treatment strategies, and the integration of basic and clinical research.

## 1. Introduction

Intussusception is one of the most common acute abdominal conditions in pediatric surgery, characterized by the telescoping of a segment of the intestine into an adjacent segment, leading to intestinal obstruction.^[1]^ It primarily affects infants and young children, particularly those aged 6 months to 2 years, with clinical manifestations including paroxysmal abdominal pain, vomiting, bloody stools, and a palpable abdominal mass.^[2]^ Without timely diagnosis and treatment, it can lead to severe complications such as intestinal necrosis, perforation, and even life-threatening conditions.^[3]^ Recent advancements in medical imaging and minimally invasive techniques have revolutionized the diagnosis and management of intussusception. Ultrasound, owing to its noninvasive nature and speed, is now the first-line diagnostic tool.^[4]^ Treatment has shifted towards less invasive procedures, with ultrasound or X-ray-guided hydrostatic or pneumatic reduction largely replacing open surgery, providing the benefits of reduced trauma and faster recovery times.^[5]^ Despite these advancements, the etiology and pathophysiology of intussusception remain incompletely understood, and recurrence remains a concern, motivating ongoing research in this area. Current research primarily focuses on case reports, mechanistic studies, and predictive modeling. Given the multifactorial nature of intussusception, a comprehensive, multidimensional approach to understanding its causes and outcomes is essential.^[6-8]^ Furthermore, there is a lack of systematic reviews summarizing the state of research in this field.

Bibliometric, as a quantitative method for analyzing scientific research output, can objectively reflect the research landscape, hotspots, and trends in a given field.^[9,10]^ The application of bibliometric techniques to medical research enables a structured, visual representation of research progression. This study seeks to use bibliometric methods to summarize the existing body of research on intussusception, identify key research themes, and outline potential future directions.

## 2. Materials and methods

### 2.1. Literature sources and search strategy

**Figure 1.**
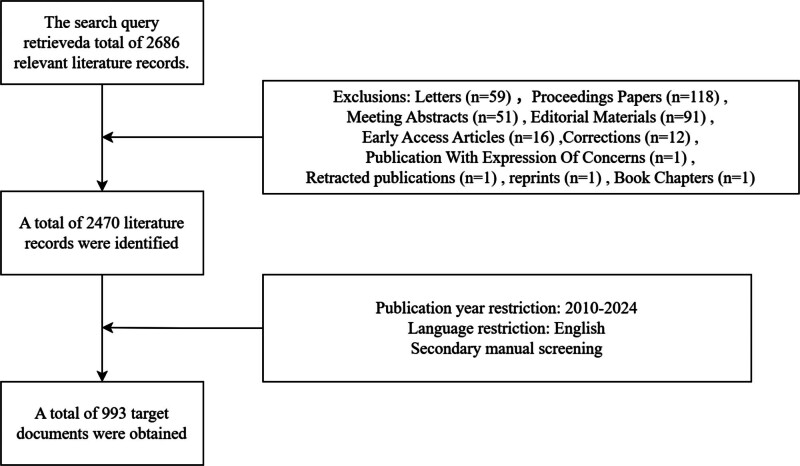
Flow chart of literature search and screening.

The Web of Science Core Collection was selected as the data source. The search query was formulated as follows: “TS = (‘intussusception’) AND (‘children’ OR ‘pediatric’ OR ‘infant’ OR ‘neonatal’ OR ‘child’ OR ‘childhood’ OR ‘toddler’ OR ‘newborn’).” The search was conducted on March 31, 2025, yielding 2686 publications. The study was limited to articles and reviews published in English between January 2010 and December 2024. Following a rigorous screening process, 993 articles were selected for further analysis (Fig. 1). To ensure comprehensive and rigorous data collection in the field of intussusception research, a supplementary search was conducted in PubMed using the following MeSH terms: (“Intussusception”[Mesh]) AND (“Child”[Mesh] OR “Pediatrics”[Mesh] OR “Infant”[Mesh]). This search, performed on July 20, 2025, initially retrieved 3504 publications. After limiting clinical studies published between January 2010 and December 2024, 118 articles were ultimately included for additional analysis.

### 2.2. Visualization and statistical analysis

All eligible articles and their citations were exported in plain text format “download_txt” and imported into CiteSpace V 6.4.R1 and VOSviewer 1.6.20 for analysis. By extracting information including authors, institutions, journals, citations, keywords, and countries/regions, this study analyzes the overall network structure, clustering patterns, key nodes, and timeline visualization in the field of intussusception research. CiteSpace adopts a set theory-based data normalization approach to measure the similarity of knowledge units. A similar algorithm is applied within time slices to generate time zone views and timeline views, producing keyword timeline maps, as well as the identification and analysis of burst terms. VOSviewer utilizes a probability theory-based data normalization method, enabling visual analysis in fields such as countries, institutions, authors, journals, and co-citation networks.^[11,12]^

## 3. Results

### 3.1. Annual growth trend of publications

**Figure 2.**
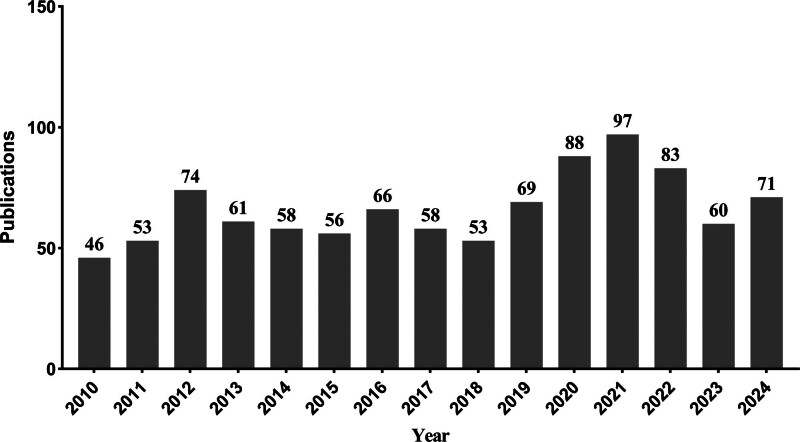
Time trend of the publications on pediatric intussusception.

After rigorous screening, 993 relevant publications were identified from 98 countries, contributed by 1580 institutions and 4891 authors. These studies were published in 288 journals and cited 13,777 references from 3876 source journals. The annual publication volume of pediatric intussusception research papers demonstrated a generally stable increasing trend over time. Specifically, the number of publications gradually increased during the period from 2010 to 2012, stabilized above 50 articles annually from 2013 to 2019, experienced substantial growth during 2020 to 2022, and continued with stable output exceeding 60 articles per year in 2023 and 2024 (Fig. 2).

### 3.2. Analysis of country/region and institution

**Figure 3.**
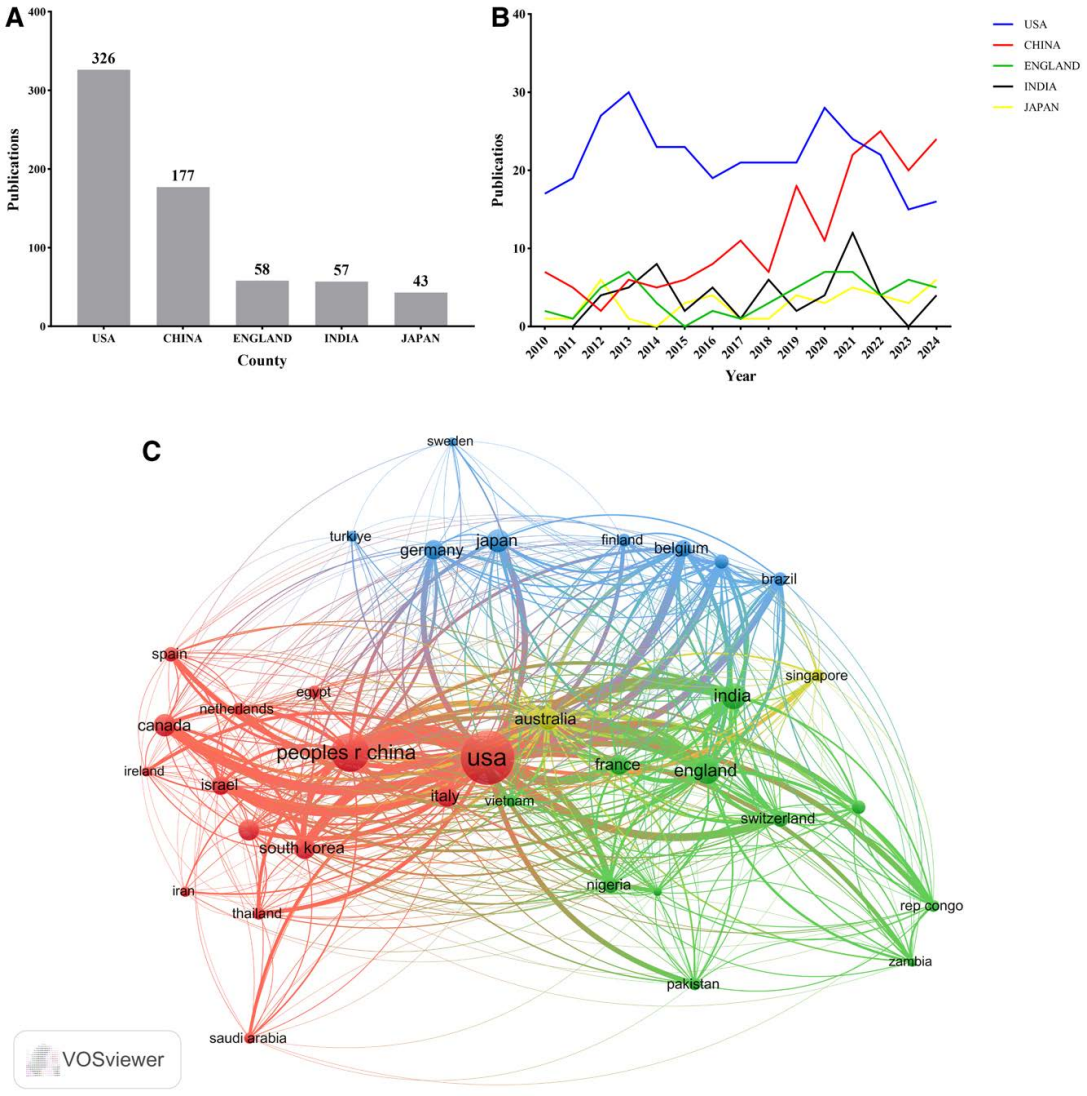
(A) Top 5 countries/regions with the largest number of publications; (B) the trend chart of the top 5 countries/regions with the largest publication output; (C) country cooperation network; (In Figure C, the node size corresponds to publication volume (larger nodes indicate higher output), connecting lines represent association strength (thicker lines denote more frequent collaborative publications between countries), and node colors distinguish different clusters^[12]^).

The study analyzed publication outputs from 98 countries/regions. Figure 3C visually presents countries with more than 5 publications using VOSviewer.

The top 5 countries contributing the most publications were the United States (326 articles, 32.8%), China (177 articles, 17.8%), the United Kingdom (58 articles, 5.8%), India (57 articles, 5.7%), and Japan (43 articles, 4.3%; Fig. 3A, B). These nations demonstrated a prominent disparity in research output, with the United States showing the most prolific contributions, followed by China. International collaboration was notably strong between these countries, especially between the United States and other leading research nations (Fig. 3C).

**Figure 4.**
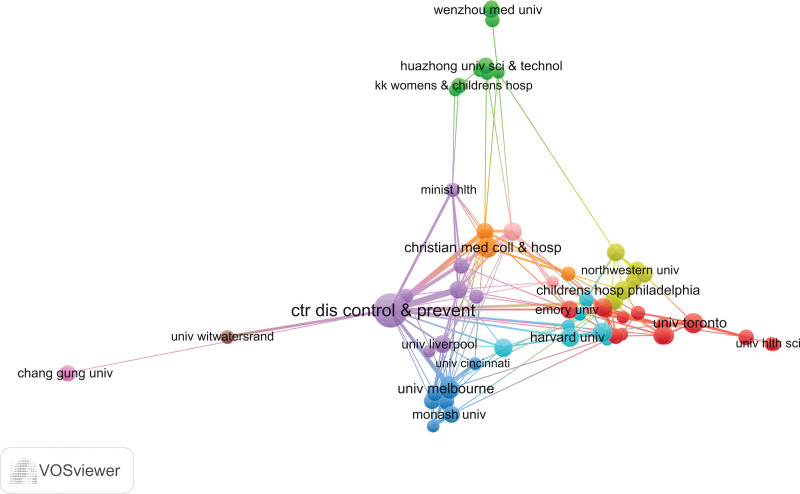
Institutional cooperation network.

Among the 1580 contributing institutions, the top 20 accounted for 28.4% of the total publication output. The US Centers for Disease Control and Prevention (CDC) was the leading institution, contributing 52 articles (5.24%). Other notable institutions included Capital Medical University from China, which ranked within the top 20 institutions with 11 articles (1.1%). Institutional collaborations, while important, were less extensive than international collaborations. The most frequent institutional collaboration was between CDC and the University of Melbourne in Australia (Fig. 4).

### 3.3. Analysis of authors

**Figure 5.**
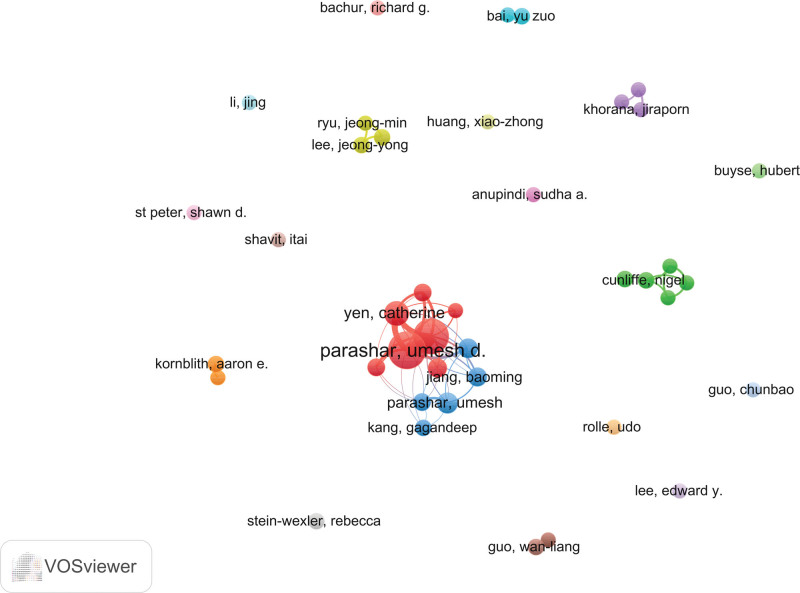
author’s cooperation network.

The co-authorship network involved 3702 authors including first and corresponding authors (Fig. 5). The most prolific author in this field was Prof Umesh D. Parashar from the CDC, with 41 publications (4.13%) and 1206 citations (29.41 citations per article). Dr Jacqueline E. Tate, also from the CDC, ranked second with 36 publications (3.95%) and 700 citations (19.44 citations per article; Table 1).

**Table 1:** Most important authors in the pediatric intussusception research field.

Rank	Author	Documents	Citations	Average citation/publication
1	Umesh D. Parashar	41	1206	29.41
2	Jacqueline E. Tate	36	700	19.44
3	Catherine Yen	15	291	19.40
4	Manish M. Patel	9	342	38
5	Jiangbaoming	8	278	34.75

### 3.4. Analysis of journals

**Figure 6.**
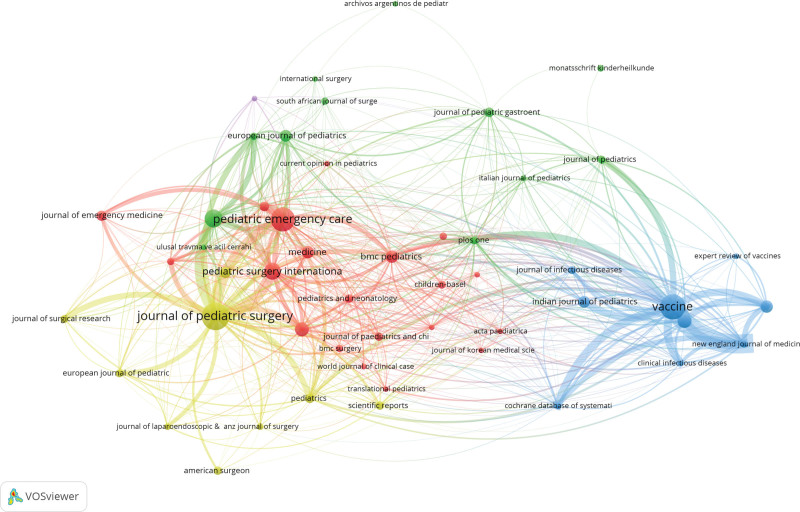
Journal’s cooperation network.

The top 3 journals by publication volume in the field of pediatric intussusception research were the *Journal of Pediatric Surgery* (61 articles), *Vaccines* (53 articles), and *Pediatric Emergency Care* (50 articles). Although Pediatric Infectious Disease Journal published fewer articles (21 articles), it had the highest citation impact, with an average of 27.90 citations per article (Table 2, Fig. 6).

**Table 2:** Top 10 journals in the pediatric intussusception research field.

Rank	Source	Publications	Citations	Average citation/publication
1	Journal of Pediatric Surgery	61	1080	17.70
2	Vaccines	53	1217	22.96
3	Pediatric Emergency Care	50	457	9.14
4	Pediatric Radiology	31	430	13.87
5	Pediatric Surgery International	28	353	12.61
6	Pediatric Infectious Disease Journal	21	586	27.90
7	Frontiers In Pediatrics	20	157	7.85
8	Human Vaccines & Immunotherapy	17	201	11.82
9	BMC Pediatrics	17	178	10.47
10	Medicine	17	102	6.00

### 3.5. Analysis of citations

**Figure 7.**
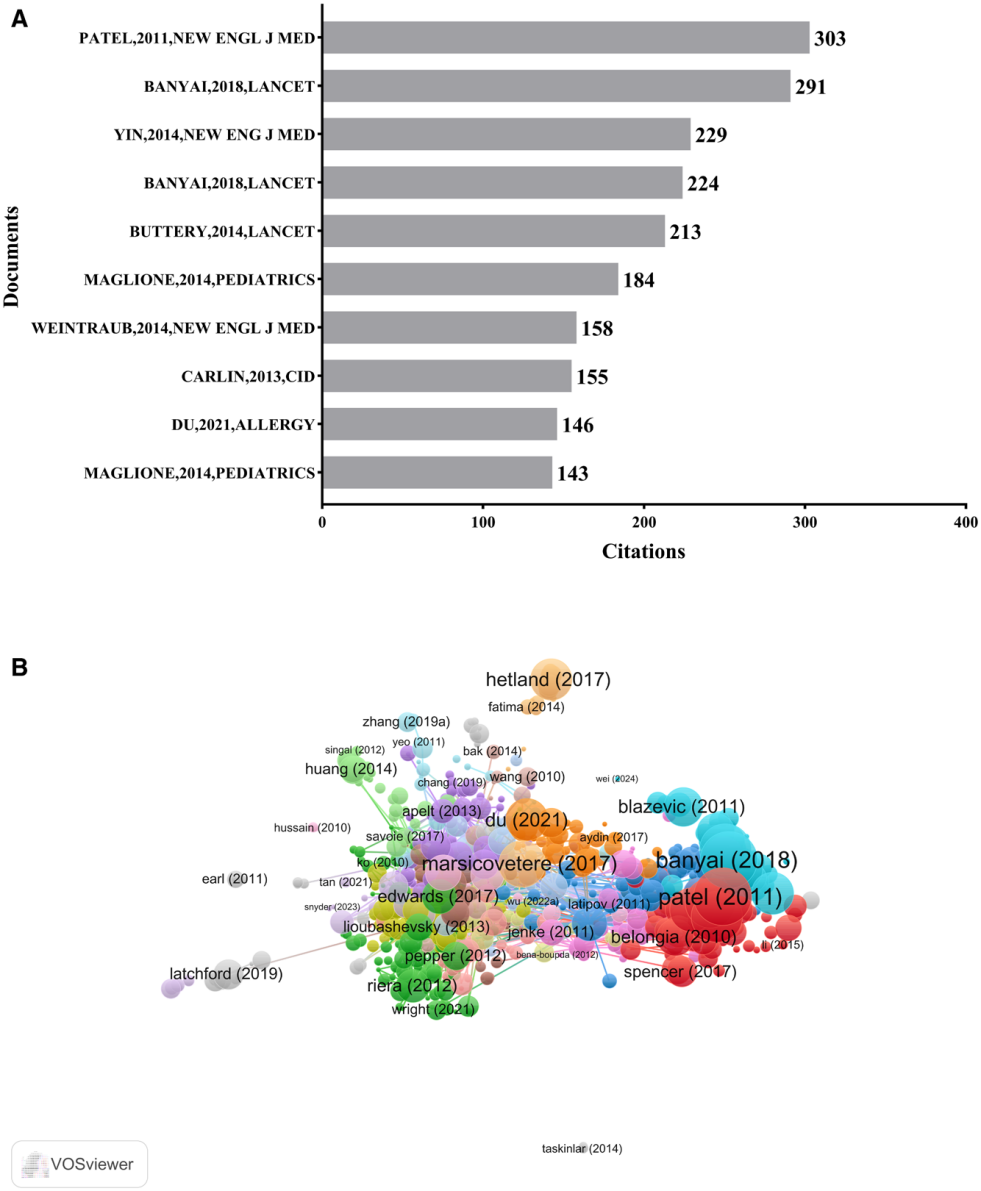
(A) Top 10 most-cited articles and (B) co-citation network.

Among the 10 most-cited articles, 7 focused on rotavirus vaccine-associated intussusception risk, employing various methodologies including retrospective studies, case-control studies, and randomized controlled trials (RCTs). Four of these articles were published in high-impact journals, such as *Lancet* (2 articles, Impact Factor [IF] = 98.4) and *New England Journal of Medicine* (2 articles, IF = 96.2). The article titled “Intussusception Risk and Health Benefits of Rotavirus Vaccination in Mexico and Brazil” published in 2011 by Prof Manish M. Patel and his research team, ranked first with a total of 303 citations (Fig. 7A, B).^[13]^

### 3.6. Analysis of keywords

**Figure 8.**
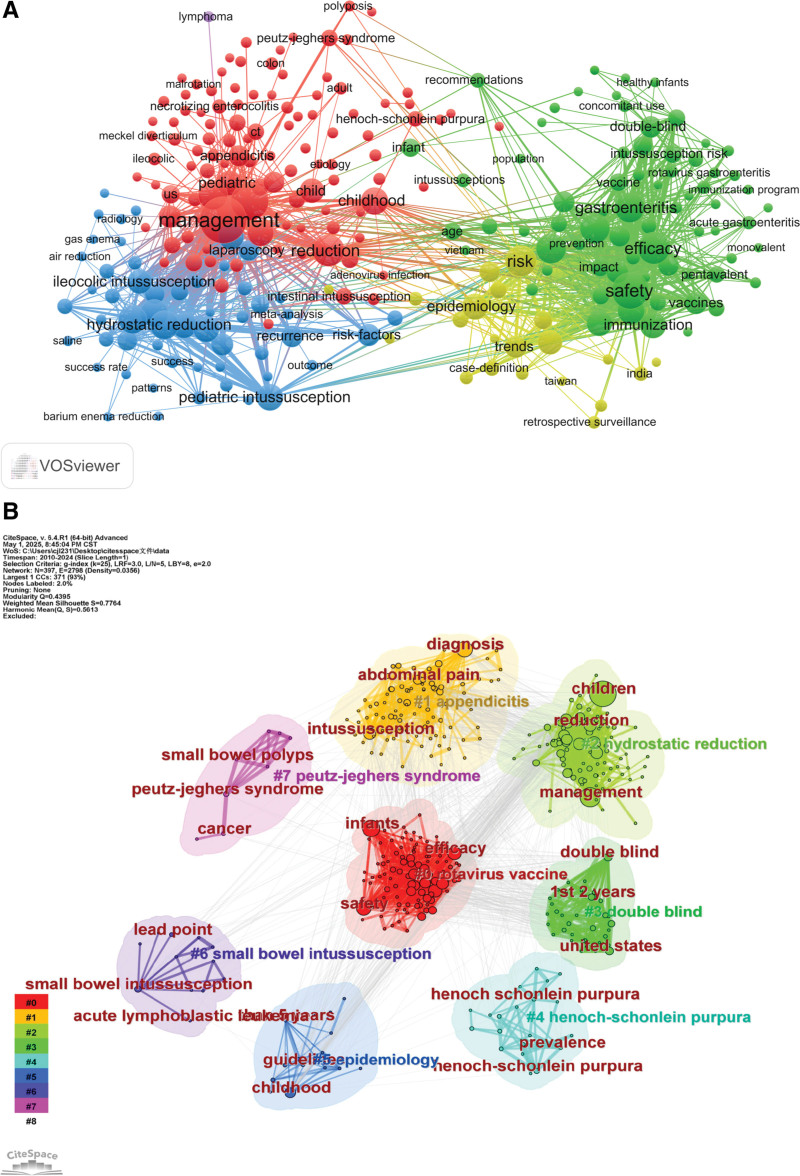
2010 to 2024 keyword analysis. (A) Keyword co-occurrence network; (B) keyword cluster analysis: (#0 Rotavirus vaccine: infants; safety; efficacy; #1 Appendicitis: diagnosis; intussusception; abdominal pain; #2 Hydrostatic reduction: children; management; reduction; #3 Double-blind: United States; double-blind; 1st 2 years; #4 Henoch–Schönlein purpura: Henoch–Schönlein purpura; Henoch–Schönlein purpura; prevalence; #5 Epidemiology: childhood; guidelines; than 5 years; #6 Small bowel intussusception: small bowel intussusception; lead point; acute lymphoblastic leukemia; #7 Peutz–Jeghers syndrome: Peutz–Jeghers syndrome; cancer; small bowel polyps).

**Figure 9.**
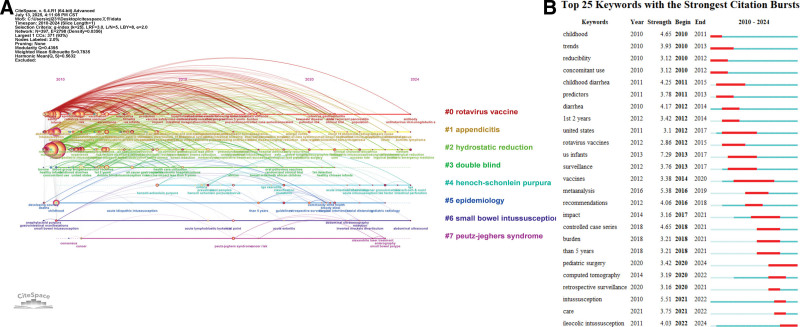
(A) Timeline chart of the keyword: #0 Rotavirus vaccine; #1 Appendicitis; #2 Hydrostatic reduction; #3 Double-blind; #4 Henoch–Schönlein purpura; #5 Epidemiology; #6 Small bowel intussusception; #7 Peutz–Jeghers syndrome; (B) The top 25 keywords with the strongest citation burst.

Keyword cluster analysis revealed 8 major research themes: rotavirus vaccine, appendicitis, hydrostatic reduction, double-blind, Henoch–Schönlein purpura, epidemiology, small bowel intussusception, and Peutz–Jeghers syndrome (Fig. 8). The timeline visualization, which incorporates both keyword co-occurrence and burst term analysis, reveals that “vaccines and child health” have constituted a core research domain, particularly focusing on the rotavirus vaccine and its association with diarrhea. Notably, the research emphasis has shifted from the traditional pathophysiology of intussusception to its clinical management and treatment outcomes (Fig. 9A, B).^[14-16]^

### 3.7. Analysis of clinical research

**Figure 10.**
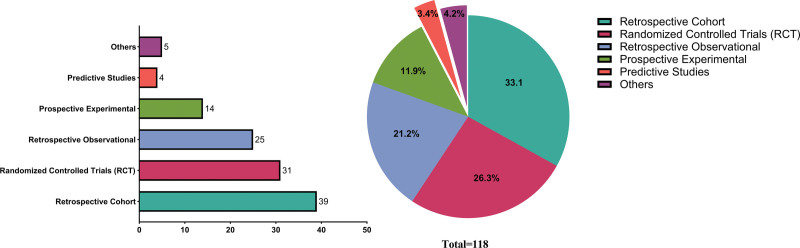
Histogram and pie diagram of clinical study types for intussusception. RCT = randomized controlled trial.

As demonstrated by the clinical research pie chart and quality assessment table, clinical research on intussusception was predominantly retrospective in nature, primarily comprising retrospective cohort studies (33.1%) and retrospective observational studies (21.2%). The research scope was relatively broad, mainly encompassing intussusception treatment, management, and diagnosis. RCTs accounted for 26.3% of studies, with their primary focus being safety evaluation of rotavirus vaccines. Prospective studies represented 11.9% of the total, while predictive studies constituted a relatively small proportion (3.4%; Fig. 10, Table S1, Supplemental Digital Content, https://links.lww.com/MD/R544).

## 4. Discussion

Over the past 15 years, the annual volume of publications on pediatric intussusception has shown a steady upward trend, suggesting that the research field is reaching a stage of dynamic equilibrium. Notably, between 2020 and 2022, there was a significant surge in publications, likely influenced by the global focus on the COVID-19 pandemic and the heightened attention on rotavirus vaccination studies during this period (Fig. 1).^[17,18]^

The geographical distribution of research outputs presents a marked disparity, with a disproportionate concentration of publications from a select number of countries. The United States has been the most prolific contributor, followed by China, the United Kingdom, India, and Japan (Fig. 3A, B and Table 3). This trend is consistent with previous studies showing that research in pediatricintussusception is predominantly driven by high-income countries, particularly those with advanced healthcare systems and research infrastructures. Additionally, the United States has emerged as the primary global leader in research output, as evidenced by both the quantity and citation frequency of its publications. Among the top 20 most productive institutions, the majority are based in the United States, with the CDC being the most influential institution in this domain. Professor Umesh D. Parashar from CDC ranks first in both publication output and citation frequency in this field, having made landmark contributions to establishing pediatric intussusception as a distinct research domain.^[19,20]^

**Table 3:** Top 5 countries/regions in the pediatric intussusception research field.

Rank	Country	Publications	Citations	Average citation/publication
1	USA	326	7111	21.81
2	CHINA	177	1365	9.55
3	ENGLAND	58	1195	20.60
4	INDIA	57	735	12.89
5	JAPAN	43	353	8.21

The *Journal of Pediatric Surgery* has maintained a central role in disseminating research on pediatric intussusception, particularly regarding surgical management, etiology, and long-term outcomes, reflecting its ongoing relevance to the clinical community.^[21,22]^ Furthermore, the substantial number of publications in *Vaccines* underscores the growing interest in the relationship between the rotavirus vaccine and the risk of intussusception, emphasizing the importance of vaccine safety surveillance and its role in shaping immunization policies globally.^[23]^

The study of surgical interventions for secondary intussusception and predictive research on recurrent cases has expanded its focus beyond the traditional under-2-year-old patient population to include children up to 5 years of age. Methodologically, the field has progressed from initial exploratory approaches (trend analysis) to more advanced evidence-based methods (meta-analyses,controlled case series), with increasing adherence to clinical practice guidelines (9). Furthermore, recent advances in imaging technology integration, particularly computed tomography (CT) scanning, have introduced novel diagnostic and therapeutic possibilities, creating new paradigms beyond conventional approaches (Fig. 9B).^[24]^

In the context of traditional intussusception pathogenesis, pediatric intussusception is associated with immature intestinal neural regulation and disrupted peristaltic rhythms in children.^[25]^ In recent years, the safety of rotavirus vaccines has become a research focus in the etiology of intussusception.^[20]^ A seminal study by Murphy et al in 1999 was the first to identify a potential association between the first-generationrotavirus vaccine and an increased risk of intussusception.^[25]^ Their findings revealed that the risk of intussusception increased by a factor of 21.7 (95% CI: 9.6–48.9) after the first dose of the vaccine, and by a factor of 3.3 (95% CI: 1.1–9.8) following the second dose. This resulted in an overall increased incidence of intussusception, ranging from 1 in 4670 to 1 in 9474 vaccinated infants. These significant findings prompted sustained efforts from both academic researchers and pharmaceutical manufacturers to improve the vaccine’s safety profile. Through iterative advancements and the development of newer vaccine generations, such as RV1 and RV5, contemporary rotavirus vaccines used in clinical practice have demonstrated a substantial reduction in the risk of intussusception.^[26-28^.

The keyword cluster map and keyword timeline analysis reveal a significant shift in the diagnostic approaches for intussusception, transitioning from reliance on clinical symptom observation to the utilization of precise, modern imaging techniques. The emergence of terms such as “childhood diarrhea” (2011–2015, strength = 4.25) and “diarrhea” (2012–2014, strength = 4.17) indicates that early research efforts were primarily focused on establishing clinical symptom-based diagnostic criteria, where the systematic evaluation of patient presentations aimed to enhance the accuracy of early diagnosis.^[29]^ Ultrasonography has become an essential first-line screening tool in standard diagnostic protocols due to its noninvasive nature and procedural convenience. Notably, the ongoing advancement of diagnostic technologies has increasingly underscored the value of imaging techniques in differential diagnosis, particularly in differentiating intussusception from other clinically similar conditions, such as appendicitis (#1) and Henoch–Schönlein purpura (#2), both of which have considerable clinical significance. The recent emergence of “CT” (2020–2022, strength = 3.19) as a key research focus reflects the growing clinical demand for accurate diagnosis in complex cases and the heightened emphasis by healthcare institutions on diagnostic precision. This trend highlights the field’s progressive shift toward more sophisticated imaging technologies in the management of intussusception (Figs. 8B and 9A).^[30,31]^

The clinical management of intussusception has undergone a significant shift from empirical treatment methods to evidence-based practices. Therapeutic research has progressively refined the core technique of hydrostatic reduction (#3), with early studies establishing its foundational role while simultaneously exploring adjunctive pharmacological therapies to develop preliminary treatment protocols. Over time, accumulated clinical experience and evidence-based methodologies, such as meta-analyses, have been employed to systematically evaluate the technical parameters, procedural standards, and therapeutic outcomes of hydrostatic reduction. This rigorous, evidence-driven approach has enabled substantial optimization and standardization of this pivotal technique. In parallel, surgical interventions have evolved into essential adjunctive options for specific conditions, such as Peutz–Jeghers syndrome (#5) and secondary small bowel intussusception (#6) of various etiologies.^[32]^ This dual development underscores a comprehensive approach to managing the diverse subtypes of intussusception.^[33]^

The analysis of burst term intensity and duration reveals distinct phase-specific characteristics in the research trends related to pediatric intussusception. The emergence of terms such as “rotavirus vaccine” (2012–2015, strength = 2.86) and “US infants” (2013–2017, strength = 7.29) reflects an early emphasis on vaccine safety surveillance. Following this, research shifted toward evidence synthesis post-2016, as evidenced by the rise of burst terms like “meta-analysis” (2016–2019, strength = 5.38) and “clinical recommendations” (2016–2018, strength = 4.06). Recent research priorities have increasingly concentrated on diagnostic and therapeutic optimization, with terms such as “case-control study” (2018–2021, strength = 4.65), “pediatric surgery” (2020–2024, strength = 3.42), and “CT” (2020–2022, strength = 3.19) emerging as cutting-edge focal points, signaling the field’s transition from epidemiological investigations to the refinement of clinical protocols. Notably, “vaccine” exhibited the longest duration of burst activity, while “US infants” demonstrated the highest burst intensity, emphasizing both the sustained importance of rotavirus vaccine research and the pivotal role of this population in studies of vaccine-associated intussusception. The burst term analysis illustrates an evolutionary trajectory from population-level monitoring to precision medicine, initially focusing on vaccine safety and epidemiological characteristics, advancing through clinical trials and evidence-based research, and ultimately converging on imaging diagnostics and individualized predictive models.^[34,35]^ This developmental pathway illustrates the discipline’s comprehensive progression from fundamental clinical observation through evidence-based practice to the current era of precision medicine (Fig. 9B).

From a quality assessment standpoint, current clinical research on intussusception exhibits distinct stratification. Retrospective studies form the core of the evidence base (55.3%), with quality scores predominantly ranging between 6 and 7, reflecting moderate-to-upper intermediate evidence levels. However, these studies commonly suffer from limitations in selection bias control and management of confounding factors. Notably, RCTs account for 26.3% of studies, with 64% rated as having low risk of bias. The use of RCT designs has significantly enhanced evidence reliability, particularly in rotavirus vaccine safety studies. In contrast, retrospective observational studies display substantial quality variability (Quality Score: 4–8), warranting cautious interpretation of their findings. Methodologically, although prospective studies and predictive model research remain limited in number, they demonstrate more rigorous design approaches. Overall, the existing evidence exhibits a “bulging middle with tapered ends” distribution pattern – medium-quality studies dominate, while high-quality RCTs and low-quality descriptive studies occupy the extremes. This distribution highlights the need for improved methodological rigor in future research, particularly through well-designed prospective cohort studies and multicenter RCTs to address the relative scarcity of high-quality evidence. To facilitate rapid case integration and minimize selection bias, retrospective studies are progressively transitioning from single center to multicenter designs. Moving forward, researchers should prioritize high-quality retrospective studies and prospective multicenter RCTs to generate more robust and clinically meaningful evidence (Fig. 10, Table 4).

**Table 4:** Distribution of clinical study types for intussusception.

Rank	Clinical study type	Publications	Ratio
1	Retrospective cohort	39	33.1
2	Randomized controlled trials (RCT)	31	26.3
3	Retrospective observational	25	21.2
4	Prospective experimental	14	11.9
5	Others	5	4.2
6	Predictive studies	4	3.4

RCT = randomized controlled trial.

Several limitations should be acknowledged in this study. First, all literature data were exclusively sourced from the Web of Science Core Collection. While this database indexes over 11,000 authoritative, high-impact international journals, offering broad coverage and robust analytical capabilities, the reliance on a single database may have led to the omission of relevant publications available in other sources. Second, the manual exclusion of articles deemed irrelevant to the research objectives may have introduced selection bias. Despite these limitations, the current study provides a comprehensive analysis of the research status and advancements in pediatric intussusception from 2014 to 2024. Future research should prioritize the following areas: elucidating the mechanisms of vaccine safety, developing advanced diagnostic tools utilizing artificial intelligence, optimizing minimally invasive treatment protocols, and establishing robust long-term follow-up systems. These efforts will be crucial in advancing pediatric intussusception care, ultimately contributing to the reduction of incidence rates and the improvement of clinical outcomes.

## 5. Conclusion

The United States and China were the most productive regions in pediatric intussusception research. The most influential country in this field is the United States, and the most influential author is Umesh D. Parashar. The focus of keyword research has gradually shifted from traditional intussusception itself to clinical management of patients. Citation analysis shows that while research methods for pediatric intussusception are relatively diverse, researchers need to vigorously conduct high-quality retrospective studies and case-control experiments to obtain higher-quality and more effective clinical evidence. Additionally, it is recommended to pay attention to promising research hotspots such as interdisciplinary diagnosis and treatment, medical-industrial integration, and medical AI.

## Author contributions

**Conceptualization:** Jialin Chen.

**Data curation:** Jialin Chen, Yong Du.

**Formal analysis:** Xiaoting Wang.

**Funding acquisition:** Ping Yang.

**Project administration:** Ping Yang.

**Resources:** Ping Yang.

**Validation:** Xiaoting Wang, Dong Xu, Yong Du.

**Visualization:** Jialin Chen.

**Writing – original draft:** Jialin Chen, Xiaoting Wang, Dong Xu.

**Writing – review & editing:** Yong Du, Ping Yang.

## Supplementary Material
