# Supplementary material for: Bibliometric analysis of research progress in pediatric intussusception from the Web of Science Core Collection over the past 15 years
Source: Medicine (Baltimore). 2026 Mar 13;105(11):e48054. doi: 10.1097/MD.0000000000048054 (PMC12991643; doi:10.1097/MD.0000000000048054)
Supplement: Supplementary file 1 [file medi-105-e48054-s001.docx]

Table S1. Quality Evaluation Table of Intussusception Studies

| Rank | First Author | year | country | Trail | Topic | Study | QS# |
| --- | --- | --- | --- | --- | --- | --- | --- |
| 1 | Vega et al. | 2015 | Spain | Others | Causes of intussusception | Caused by recent medications | ~ |
| 2 | Shekherdimian et al. | 2011 | America | Retrospective Cohort | Management of intussusception | Differences in the management of children with intussusception at different ages | 7 |
| 3 | Adekunle-Ojo et al. | 2011 | America | Retrospective Cohort | Management of intussusception | Effect of feeding time after intussusception reduction | 7 |
| 4 | Stone et al. | 2013 | America | Retrospective Cohort | Management of intussusception | Incidence of complications after intussusception surgery | 7 |
| 5 | Klein et al. | 2013 | America | Retrospective Cohort | Management of intussusception | Risk factors after intussusception surgery | 7 |
| 6 | Curtis et al. | 2010 | America | Retrospective Cohort | Management of intussusception | Impact of treatment of intussusception in non-pediatric hospitals versus pediatric hospitals | 5 |
| 7 | Niramis et al. | 2010 | Thailand | Retrospective Cohort | Management of intussusception | Management strategy for recurrent intussusception | 7 |
| 8 | Beres et al. | 2014 | Canada | Retrospective Cohort | Management of intussusception | Difference between emergency management and hospitalization | 7 |
| 9 | Whitehouse et al. | 2010 | America | Retrospective Cohort | Management of intussusception | Management of discharge without observation after successful water enema | 4 |
| 10 | Kodikara et al. | 2010 | New Zealand | Retrospective Observational | Management of intussusception | Differences in intussusception management in different populations | 6 |
| 11 | Jehangir et al. | 2014 | India | Retrospective Observational | Management of intussusception | Difference between active and passive monitoring | 6 |
| 12 | Delgado et al. | 2021 | China | Retrospective Observational | Management of intussusception | Ultrasound benefit | 7 |
| 13 | Rattan et al. | 2016 | India | Retrospective Observational | Management of intussusception | Management of secondary intussusception | 5 |
| 14 | England et al. | 2012 | South Africa | Retrospective Observational | Management of intussusception | Clinical signs of secondary intussusception | 6 |
| 15 | Kwon et al. | 2019 | Korea | Retrospective Observational | Management of intussusception | Length of hospital stay vs. recurrence | 6 |
| 16 | Fiordaliso et al. | 2021 | Italy | Others | Management of intussusception | Differences in clinical manifestations of intussusception |  |
| 17 | Chen et al. | 2010 | China | Others | Management of intussusception | Management of secondary intussusception | ~ |
| 18 | Sujka et al. | 2019 | America | Prospective Experimental | Management of intussusception | Emergency observation and early discharge | 5 |
| 19 | Freedman et al. | 2014 | Canada | Retrospective Cohort | Epidemiology of intussusception | Misdiagnosis rate and influencing factors | 7 |
| 20 | Peyvasteh et al. | 2011 | Iran | Retrospective Cohort | Epidemiology of intussusception | Intussusception and intestinal obstruction | 6 |
| 21 | Ortegón et al. | 2022 | Mexico | Retrospective Observational | Epidemiology of intussusception | Epidemiological characterization analysis | 4.5 |
| 22 | Mattei et al. | 2017 | Italy | Retrospective Observational | Epidemiology of intussusception | Intussusception versus acute gastroenteritis | 7 |
| 23 | Hwang et al. | 2019 | Spain | Retrospective Observational | Epidemiology of intussusception | Incidence of intussusception | 6 |
| 24 | Fotso et al. | 2017 | France | Prospective Experimental | Epidemiology of intussusception | Incidence of intussusception | 8 |
| 25 | Sáez-Llorens et al. | 2013 | Panama | Prospective Experimental | Epidemiology of intussusception | Latin American Intussusception Epidemiology | 8 |
| 26 | Zhang et al. | 2011 | China | Retrospective Cohort | Intussusception prediction | Surgical prediction of intussusception of small bowel by ultrasound | 7 |
| 27 | Territo et al. | 2014 | America | Retrospective Observational | Intussusception prediction | Relationship between intussusception clinical manifestations and recurrence | 7 |
| 28 | Weihmiller et al. | 2011 | America | Retrospective Observational | Intussusception prediction | Decision Tree Prediction Model | 8 |
| 29 | Ademuyiwa et al. | 2018 | Nigeria | Prospective Experimental | Intussusception prediction | Laboratory tests predict intestinal necrosis | 6 |
| 30 | Zhu et al. | 2022 | China | Predictive Studies | Intussusception prediction | Effect of MCP-1 on intussusception recurrence | Low risk of bias |
| 31 | Guo et al. | 2022 | China | Predictive Studies | Intussusception prediction | Recurrence prediction model | Moderate risk of bias |
| 32 | Henderson et al. | 2013 | America | Retrospective Cohort | Intussusception diagnosis | Screening performance of X-ray and ultrasound | 7 |
| 33 | Chang et al. | 2013 | China | Retrospective Cohort | Intussusception diagnosis | Difference between daytime and nighttime imaging diagnosis of intussusception | 6 |
| 34 | Usang et al. | 2013 | Nigeria | Retrospective Cohort | Intussusception diagnosis | Accuracy of ultrasound in developing countries | 6 |
| 35 | Aronson et al. | 2013 | America | Retrospective Cohort | Intussusception diagnosis | Consistency between emergency and radiologist readings | 7 |
| 36 | Park et al. | 2019 | Belgium | Retrospective Cohort | Intussusception diagnosis | Distinction in imaging features | 6 |
| 37 | Newman et al. | 2014 | America | Retrospective Observational | Intussusception diagnosis | Imaging differentiation between intussusception and appendicitis | 5 |
| 38 | Mendez et al. | 2012 | America | Retrospective Observational | Intussusception diagnosis | Diagnostic accuracy of ultrasound and x-ray | 7 |
| 39 | Zhang et al. | 2016 | China | Retrospective Observational | Intussusception diagnosis | Diagnosis of secondary intussusception by ultrasound | 6 |
| 40 | Bergmann et al. | 2021 | America | Retrospective Observational | Intussusception diagnosis | Diagnostic value of point-of-care ultrasound | 8 |
| 41 | Rajagopal et al. | 2015 | India | Retrospective Observational | Intussusception diagnosis | Ultrasound features of intussusception Requiring surgical reduction | 5 |
| 42 | Arroyo et al. | 2021 | America | Prospective Experimental | Intussusception diagnosis | Emergency ultrasound value | 6 |
| 43 | Savoie et al. | 2017 | America | Retrospective Cohort | Intussusception treatment | Treatment of children over 3 years of age | 7 |
| 44 | Nguyen et al. | 2024 | Vietnam | Retrospective Cohort | Intussusception treatment | Success Rate of Intussusception Modified Air Enema | 8 |
| 45 | Hill et al. | 2013 | America | Retrospective Cohort | Intussusception treatment | Difference between intussusception laparoscopic surgery and open surgery | 7 |
| 46 | Sharp et al. | 2013 | America | Retrospective Cohort | Intussusception treatment | Difference between bowel resection and manual reduction in intussusception surgery | 7 |
| 47 | Esposito et al. | 2015 | Italy | Retrospective Cohort | Intussusception treatment | Non-surgical treatment difference | 6 |
| 48 | Hsu et al. | 2012 | China | Retrospective Cohort | Intussusception treatment | Time selection for recurrent intussusception surgery | 7 |
| 49 | Golriz et al. | 2018 | France | Retrospective Cohort | Intussusception treatment | Air enema balloon safety | 6 |
| 50 | Kao et al. | 2010 | China | Retrospective Cohort | Intussusception treatment | Difference in treatment of intussusception between laparoscopic versus laparotomy | 5 |
| 51 | Eraki et al. | 2017 | Egypt | Retrospective Cohort | Intussusception treatment | Water enema safety | 6 |
| 52 | Sanchez et al. | 2015 | America | Retrospective Cohort | Intussusception treatment | Complications of water versus air enema | 6 |
| 53 | Ocal et al. | 2014 | Turkey | Retrospective Cohort | Intussusception treatment | Water enema vs. surgery | 6 |
| 54 | Renzo et al. | 2012 | Italy | Retrospective Cohort | Intussusception treatment | Differences between different enemas for intussusception in special locations | 7 |
| 55 | Lampl et al. | 2019 | America | Retrospective Cohort | Intussusception treatment | Effect of Delayed Enema on Surgery | 5 |
| 56 | Ekenze et al. | 2011 | Nigeria | Retrospective Cohort | Intussusception treatment | Effect of delayed visits on intussusception | 6 |
| 57 | Al-Tokhais et al. | 2012 | Canada | Retrospective Cohort | Intussusception treatment | Effect of prophylactic antibiotics on intussusception reduction | 6 |
| 58 | Cardenal et al. | 2019 | America | Retrospective Cohort | Intussusception treatment | Long-course intussusception water enema | 6 |
| 59 | Tareen et al. | 2011 | Ireland | Retrospective Cohort | Intussusception treatment | Effect of Long Course on Air Enema Success Rate | 7 |
| 60 | Purnomo et al. | 2024 | Indonesia | Retrospective Cohort | Intussusception treatment | Sedation versus non-sedation sewer enema success rate | 7 |
| 61 | Vujović et al. | 2014 | Serbia | Retrospective Cohort | Intussusception treatment | Effect of Symptom Duration on Water Enema Success Rate | 7 |
| 62 | Lautz et al. | 2015 | America | Retrospective Cohort | Intussusception treatment | Difference in the effectiveness of treatment modalities | 8 |
| 63 | Wang et al. | 2019 | America | Retrospective Observational | Intussusception treatment | Intussusception surgery combined with appendix surgery | 6 |
| 64 | Hannon et al. | 2014 | England | Retrospective Observational | Intussusception treatment | Factors influencing reduction therapy | 6 |
| 65 | Lin et al. | 2017 | China | Retrospective Observational | Intussusception treatment | Secondary intussusception treatment | 5 |
| 66 | Lim et al. | 2018 | Indonesia | Retrospective Observational | Intussusception treatment | Water enema safety | 6 |
| 67 | Pazo et al. | 2010 | America | Retrospective Observational | Intussusception treatment | Factors affecting delayed repeat enema | 7 |
| 68 | Vazquez et al. | 2012 | Spain | Prospective Experimental | Intussusception treatment | Safety of external manual reduction for intussusception | 6 |
| 69 | Khalid et al. | 2012 | India | Prospective Experimental | Intussusception treatment | Impact of ultrasound on emergency intussusception | 7 |
| 70 | Zhang et al. | 2020 | Korea | Prospective Experimental | Intussusception treatment | Treatment of recurrent intussusception | 7 |
| 71 | Liu et al. | 2021 | Spain | Prospective Experimental | Intussusception treatment | Comparison of non-surgical reduction methods | 8 |
| 72 | Mensah et al. | 2011 | Ghana | Prospective Experimental | Intussusception treatment | Safety of air enema | 6 |
| 73 | Aldagalán et al. | 2012 | Spain | Prospective Experimental | Intussusception treatment | Efficacy of air enema under general anesthesia | 7 |
| 74 | Belsha et al. | 2017 | England | Prospective Experimental | Intussusception treatment | Role of digestive endoscopy in intussusception | 5 |
| 75 | Li et al. | 2021 | China | RCT | Intussusception treatment | Safety of non-surgical reduction | Moderate risk of bias |
| 76 | Chukwu et al. | 2023 | Nigeria | RCT | Intussusception treatment | Comparison of non-surgical reduction methods | Moderate risk of bias |
| 77 | Lian et al. | 2024 | China | RCT | Intussusception treatment | Comparison of success rates between air enema and hydrostatic enema | Moderate risk of bias |
| 78 | Eisapour et al. | 2015 | Iran | RCT | Intussusception treatment | Effect of midazolam on hydrostatic enema | Low risk of bias |
| 79 | Jamshidi et al. | 2022 | Iran | RCT | Intussusception treatment | Comparison of complications between surgical methods | Moderate risk of bias |
| 80 | Xie et al. | 2018 | China | RCT | Intussusception treatment | Comparison between hydrostatic enema and air enema | Moderate risk of bias |
| 81 | Bucher et al. | 2011 | America | Predictive Studies | Intussusception treatment | Difference between conditional contrast enema and simple enema |  |
| 82 | Hoffman et al. | 2018 | Australia | Retrospective Cohort | Rotavirus vaccine | Short-term safety of RV1 vaccine | 6 |
| 83 | Quinn et al. | 2014 | Australia | Retrospective Cohort | Rotavirus vaccine | Association between rotavirus vaccine (RV1) and intussusception | 7 |
| 84 | Loughlin et al. | 2012 | America | Retrospective Cohort | Rotavirus vaccine | Association between pentavalent rotavirus vaccine and intussusception | 7 |
| 85 | Kim et al. | 2017 | Korea | Retrospective Observational | Rotavirus vaccine | Safety of BRV-PV vaccine regarding intussusception | 6 |
| 86 | Forrest et al. | 2017 | England | Retrospective Observational | Rotavirus vaccine | Impact of rotavirus vaccine on intussusception hospitalization rates | 6 |
| 87 | Uhlig et al. | 2014 | England | Retrospective Observational | Rotavirus vaccine | Safety of rotavirus vaccine in outpatient settings | 6 |
| 88 | Vilar et al. | 2015 | Spain | Retrospective Observational | Rotavirus vaccine | Association between rotavirus and intussusception risk | 7 |
| 89 | Rosillon et al. | 2015 | Mexico | Others | Rotavirus vaccine | Intussusception risk within 7 days of RV1 and RV5 vaccination | ~ |
| 90 | Hai et al. | 2021 | Vietnam | Others | Rotavirus vaccine | Safety and immunogenicity |  |
| 91 | Weintraub et al. | 2014 | America | Prospective Experimental | Rotavirus vaccine | Association between monovalent rotavirus vaccine (Rotarix) and intussusception risk | 8 |
| 92 | Fernandes et al. | 2016 | Brazil | Prospective Experimental | Rotavirus vaccine | Epidemiology of intussusception before and after rotavirus vaccine introduction | 5 |
| 93 | Gillard et al. | 2019 | America | RCT | Rotavirus vaccine | Safety of combined/spaced administration of DPT-IPV and Rotarix | Moderate risk of bias |
| 94 | Tanaka et al. | 2017 | Japan | RCT | Rotavirus vaccine | Safety of combined administration of DTaP-sIPV and RV5 | Moderate risk of bias |
| 95 | Xia et al. | 2020 | China | RCT | Rotavirus vaccine | Safety of LLR3 vaccine | Moderate risk of bias |
| 96 | Buyse et al. | 2014 | Belgium | RCT | Rotavirus vaccine | Intussusception risk of Rotarix vaccine | Low risk of bias |
| 97 | Lau et al. | 2013 | China | RCT | Rotavirus vaccine | Safety of Rotarix vaccine in children under 3 years old | Low risk of bias |
| 98 | Coldiron et al. | 2018 | America | RCT | Rotavirus vaccine | Long-term safety of RotaSIIL vaccine | Low risk of bias |
| 99 | Suman et al. | 2022 | India | RCT | Rotavirus vaccine | Safety comparison between Rotavac and Rotasiil | Moderate risk of bias |
| 100 | Dang et al. | 2012 | Viet Nam | RCT | Rotavirus vaccine | Safety of Rotavin-M1 vaccine | Low risk of bias |
| 101 | Parra et al. | 2024 | Mexico | RCT | Rotavirus vaccine | Differences in safety between RV1 and RV5 | Low risk of bias |
| 102 | Byars et al. | 2018 | Malaysia | RCT | Rotavirus vaccine | Safety of RV3-BB vaccine in neonates/infants | Low risk of bias |
| 103 | Mo et al. | 2017 | China | RCT | Rotavirus vaccine | Risk of intussusception with RV5 vaccine in Chinese infants | Low risk of bias |
| 104 | Chilengi et al. | 2021 | India | RCT | Rotavirus vaccine | Comparison of safety among different rotavirus vaccines | Moderate risk of bias |
| 105 | Bhandari et al. | 2014 | India | RCT | Rotavirus vaccine | Safety of monovalent human-bovine reassortant rotavirus vaccine (116E) in India | Low risk of bias |
| 106 | John et al. | 2014 | India | RCT | Rotavirus vaccine | Association between oral monovalent rotavirus vaccine (116E strain) and intussusception risk | Low risk of bias |
| 107 | Bhandari et al. | 2014 | India | RCT | Rotavirus vaccine | Management of intussusception in children under 2 years old with oral rotavirus vaccine 116E | Low risk of bias |
| 108 | Thiem et al. | 2021 | Vietnam | RCT | Rotavirus vaccine | Comparison between two rotavirus vaccines (liquid vs. frozen) | Moderate risk of bias |
| 109 | Wu et al. | 2022 | China | RCT | Rotavirus vaccine | Safety of hexavalent rotavirus vaccine in Chinese children | Low risk of bias |
| 110 | Isanaka et al. | 2017 | France | RCT | Rotavirus vaccine | Safety of rotavirus vaccines | Low risk of bias |
| 111 | O'Ryan et al. | 2015 | Chile | RCT | Rotavirus vaccine | Immunogenicity and safety of rotavirus vaccines | Low risk of bias |
| 112 | Bianca et al. | 2022 | Australia | RCT | Rotavirus vaccine | Safety of rotavirus vaccines in Australian children | Low risk of bias |
| 113 | Christie et al. | 2010 | Jamaica | RCT | Rotavirus vaccine | Safety of pentavalent rotavirus vaccine | Low risk of bias |
| 114 | Christie et al. | 2012 | Jamaica | RCT | Rotavirus vaccine | Safety of pentavalent rotavirus vaccine | Low risk of bias |
| 115 | Laserson et al. | 2012 | Kenya | RCT | Rotavirus vaccine | Safety of pentavalent rotavirus vaccine in children from Asian and African countries | Low risk of bias |
| 116 | Armah et al. | 2013 | Ghana | RCT | Rotavirus vaccine | Safety of RRV-TV vaccine administration in neonates | Low risk of bias |
| 117 | Zickafoose et al. | 2012 | America | RCT | Rotavirus vaccine | Relationship between rotavirus vaccine introduction and intussusception hospitalization rates | Low risk of bias |
| 118 | Bravo et al. | 2014 | Philippines | Predictive Studies | Rotavirus vaccine | Safety and tolerability of rotavirus vaccines | ~ |

#: Quality Score (QS) represents the quality assessment score, with retrospective and prospective studies evaluated using the Newcastle-Ottawa Scale (NOS), while randomized controlled trials (RCTs) were assessed using the Cochrane Risk of Bias Tool.
